# Inhibitory Effect of Selected Guaianolide and Germacranolide Sesquiterpene Lactones on Nitric Oxide Production

**DOI:** 10.3390/molecules29143289

**Published:** 2024-07-11

**Authors:** Juraj Harmatha, Zdeněk Zídek, Eva Kmoníčková

**Affiliations:** 1 Institute of Organic Chemistry and Biochemistry, Czech Academy of Sciences, 166 10 Prague, Czech Republic; 2 Institute of Experimental Medicine, Czech Academy of Sciences, 142 20 Prague, Czech Republiceva.kmonickova@lfmotol.cuni.cz (E.K.); 3 Department of Pharmacology, Second Faculty of Medicine, Charles University, 150 00 Prague, Czech Republic

**Keywords:** guaianolides, germacranolides, immune-modulatory effects, 8-epiisoamberboin, 8-deoxylactucin

## Abstract

Trilobolide and its analogues belong to the guaianolide type of sesquiterpene lactones, which are characteristic and widely distributed within the families Asteraceae and Apiaceae. Certain guaianolides are receiving continuously increasing attention for their promising sarco-endoplasmic reticulum Ca^2+^-ATPase (SERCA)-inhibitory activity. However, because of their alkylation capabilities, they are generally toxic. Therefore, the search for compounds with significant immunobiological properties but with decreased cytotoxicities suitable for use in immune-based pharmacotherapy is ongoing. Therefore, we extended our previous investigation of the immunobiological effects of trilobolide to a series of structurally related guaianolides and germacranolides. To evaluate the relationship, we tested a series of selected derivatives containing α-methyl lactone or exomethylene lactone ring. For a wider comparison, we also included some of their glycosidic derivatives. We assessed the in vitro immunobiological effects of the tested compounds on nitric oxide (NO) production, cytokine secretion, and prostaglandin E2 (PGE2) release by mouse peritoneal cells, activated primarily by lipopolysaccharide (LPS), and evaluated their viability. The inhibitory effects of the apparently most active substance, 8-deoxylactucin, seem to be the most promising.

## 1. Introduction

The great structural diversity of sesquiterpene lactones [1,2] and the corresponding variety of their biological and pharmacological effects [3] have attracted the attention of many scientific teams around the world. The initial research on this group of compounds mainly focused on determining their chemical structures [4,5] and ascertaining their mutual phylogenetic relationships [6]. The phylogenetic relationships of the different sesquiterpene lactones were utilized for the purposes of chemosystematics, where individual substances are regarded primarily as objective chemotaxonomic characteristics [7].

**Figure 1:**
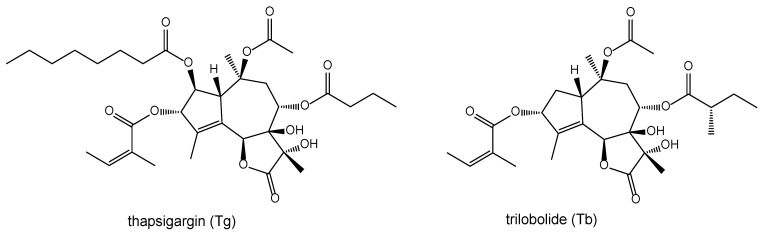
Structures of thapsigargin (Tg) and trilobolide (Tb).

We were equally interested in the biological effects of sesquiterpene lactones, especially because of their anti-feeding effect on insects [8,9], in relation to their role in the chemoecological relationship between plants and insects [10,11]. The known significant pharmacological effects of thapsigargin (Tg) [12,13] led us to also investigate the immuno-modulating activities of its structurally related natural analogue trilobolide (Tb) [14,15,16] and its selectively prepared derivatives [17,18,19]. Our intention was to explain the influence of the characteristic structurally arranged lactone group moiety of trilobolide (Tb), and especially its diol substitutions at positions 7 and 11 (Figure 1), on the expression of nitric oxide and cytokines of both thapsigargin (Tg) and trilobolide (Tb). It is remarkable, moreover, that some of their derivatives have been found to exhibit reduced toxicity [13,18].

The majority of natural sesquiterpene lactones contain their lactone ring annelated with the basic skeleton either in a *trans* or *cis* conformation. Moreover, the methyl group on the lactone ring can occur in both structural *S* or *R* (alpha or beta) configurations. The α-methylene-γ-lactone seems to play a crucial role in most of the biological activities of sesquiterpene lactones including their immunomodulatory/anti-inflammatory activity, measured as the inhibition of nitric oxide (NO) production/expression of inducible nitric oxide synthase, cytokine secretion, or prostaglandin E2 production.

However, many sesquiterpene lactones have a specific exomethylene group present in their lactone moiety, featuring substantive alkylating properties and a resultant specific biological activity. For this study, we therefore selected a series of lactones of both types (i.e., containing a methyl or an exomethylene group) so that they could be compared with two particularly effective hydroxylated lactones of the guaianolide type, trilobolide (Tb) and thapsigargin (Tg), for the modulation of pro-inflammatory markers.

**Figure 2:**
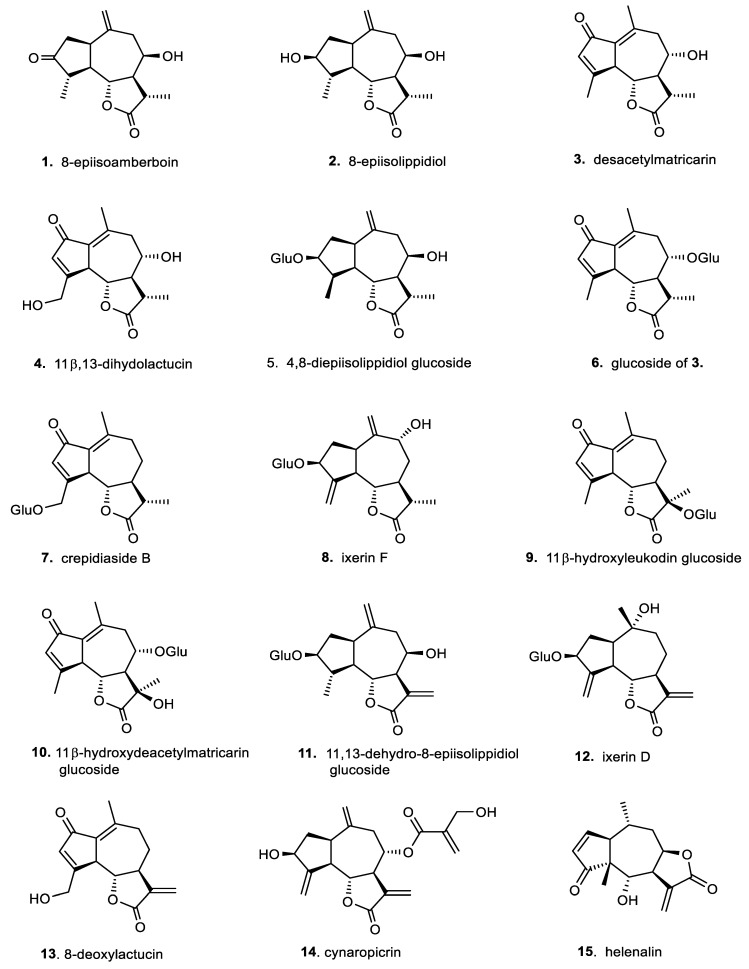
Chemical structures of sesquiterpene lactones (**1**–**10**) and related α-exomethylene lactones (**11**–**15**) of guaianolide type.

**Figure 3:**
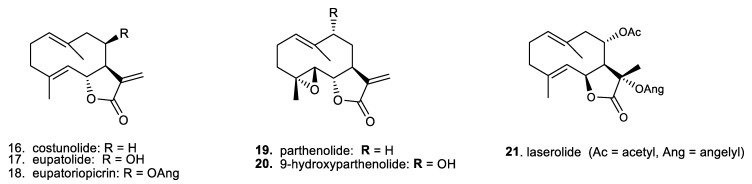
Chemical structures of biogenetically related sesquiterpene lactones of the germacranolide type, regarded as biogenetic precursors of guaianolides.

In addition, for a broader comparison of their biological effects, we also included a small series of germacranolides, which are the phylogenetic precursors of the primarily investigated guaianolides [6,12]. Overviews of the selected structural types of sesquiterpene lactones are presented in Figure 2 (for the guaianolides) and Figure 3 (for the germacranolides).

We can conclude that Tb and Tg possess distinct immunomodulatory profiles, as was described earlier [14,15]. In the groups of tested methyl and exomethylene lactones, we verified the toxicity (viability) of several compounds.

## 2. Results

### 2.1. Determination of Viability

**Figure 4:**
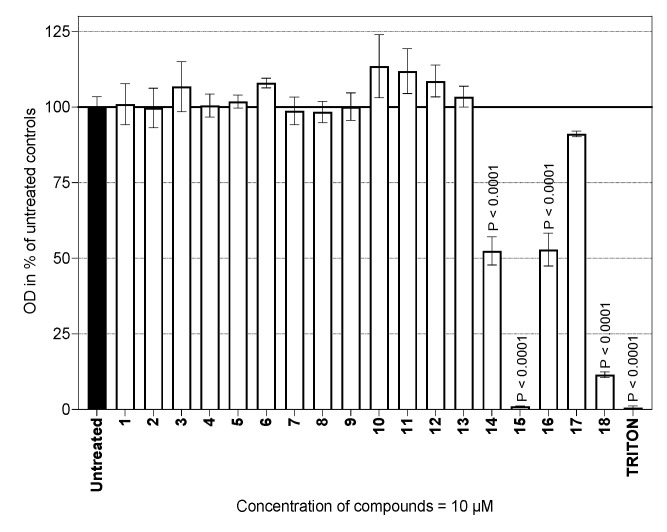
Viability of mouse peritoneal cells in the presence of the compounds tested. The cells (1.0 × 10^6^/mL) were cultured for 24 h. The effect of each compound on cell viability is expressed in percentages relative to that of untreated controls (100% viability). The columns represent means, with S.E.M. values indicated by error bars. The data originated from two experiments, each run in triplicate.

A series of two types of sesquiterpene lactones, guaianolides (Figure 2) and germacranolides (Figure 3), were screened for possible cytotoxicity. These contained either a methyl group (Figure 2, compounds **1**–**10**) or an activated exomethylene group (Figure 2 and Figure 3, compounds **11**–**20**). In the germacranolide series, mostly exomethylene lactones (Figure 3, compounds **16**–**20**) were tested, along with one specific derivative containing an angeloyl substituent on the lactone ring (Figure 3, compound **21**). Compound **21** was among the most active substances in our previous study [16,20]; however, it was tested on the cells from a different organism. The results of our current cytotoxicity assay of our main test compounds (**1**–**18**) are presented in Figure 4.

The viability of the primary murine macrophages was tested in the presence of 10 µM of each test substance. The results did not indicate any changes in viability for compounds **1–13** in comparison with that of the untreated cells. The obtained results also showed that the glucoside group did not play a role in the viability of the cells used. On the other hand, exomethylene lactones, such as cynaropicrin (**14**) and mainly helenalin (**15**), were cytotoxic (*p* < 0.0001 for both sesquiterpenes), which is consistent with the study on RAW264.7 cells by Matsumoto [21]. Based on the literature data, cynaropicrin (**14**) was found to be more cytotoxic and pro-apoptotic toward cancer cells, mainly leucocyte-derived cancer cells, compared to macrophages [22].

Among the six germacranolides in this study (**16**–**21**), two substances exhibited significant cytotoxicity: Costunolide (**16**) showed moderate toxicity, similar to cynaropicrin (**14**), the O.D. of which was approximately 50% of that of the untreated cells. The mild to moderate toxicity of costunolide (**16**) found in our study on primary murine macrophages is comparable to that in published results [23], where the macrophage cell line RAW264.7 was used as the model. Another substance studied, eupatoriopicrin (**18**), was highly toxic (O.D. of approx. 10% of that of the untreated cells) toward primary murine macrophages. A comparative study [24] demonstrated the cytotoxic effect of eupatoriopicrin extracts on RAW264.7 cells and on different cancer cells lines. In a model utilizing polymorphonuclear lymphocytes, eupatoriopicrin (**18**) was found not to be toxic only up to a concentration of 2.5 µM [25]. Our result showing the cytotoxicity of **18** thus contributes a piece to the mosaic of information on this compound. In addition to its described apoptotic and anti-inflammatory properties, changes in cellular energetic metabolism suggest that **18** is an antiparasitic agent [26]. On the other hand, none of the other germacranolides tested significantly affected cell viability. It seems that the hydroxylation of costunolide (**16**) at position C-8 makes the compound non-cytotoxic, because the viability of cells treated with eupatolide (**17**) was comparable to that of untreated cells (Figure 4). Additional experiments with substances **19**–**21** revealed, that the parthenolide compounds **19** and **20** slightly reduced cell viability to 80% of that of the untreated controls (81.14 ± 6.34 and 76.97 ± 5.09, respectively; these values being averages from two independent experiments in a duplicate setup). The pilot experiment with 10 µM laserolide (**21**) did not show any cytotoxicity in murine peritoneal cells. This is consistent with our previous results obtained with a rat macrophage model [16,19].

### 2.2. Nitric Oxide Production

Inducible nitric oxide synthase (iNOS) takes part in the production of NO in immune and other types of cells. The overproduction of NO mediates various pathophysiological problems such as acute and chronic inflammation, diabetes, septic shock, neurodegenerative damage, and autoimmune diseases. Therefore, to regulate/decrease the overproduction of NO is a persistent idea for many pharmacologists. Measuring the NO levels produced by immune cells is a standard approach to screening for compounds with possible immunomodulatory and anti-inflammatory activities. The suppression of LPS-induced NO synthesis by sesquiterpene lactones has already been well documented in many studies, e.g., [21,27]. The idea is based on the observation that the α-methylene-γ-lactone moiety might be essential for the NO inhibitory activity [27].

**Figure 5:**
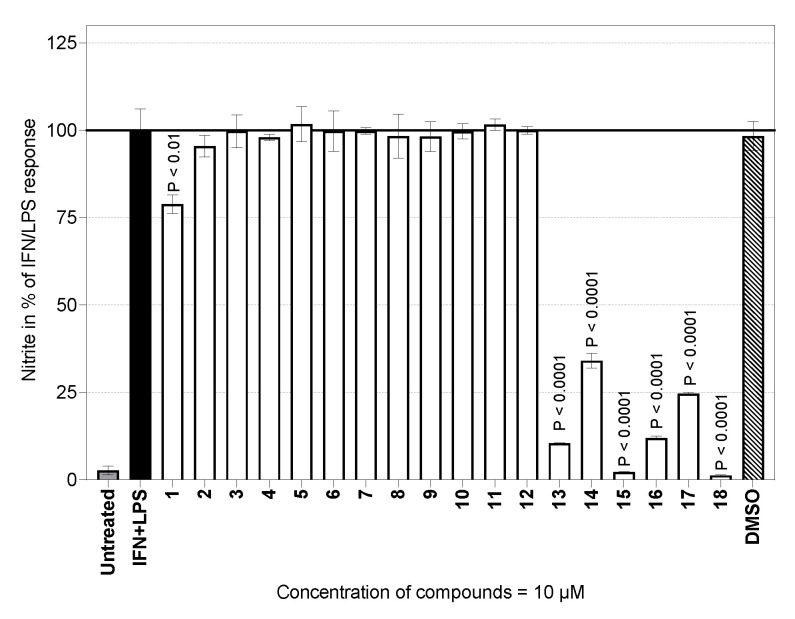
Effects of the compounds tested on the NO production by mouse peritoneal cells. The cells (2.0 × 10^6^/mL) were cultured for 24 h. The effect is expressed in percentages relative to values of controls stimulated with the IFN-γ + LPS cocktail. The columns represent means, with S.E.M. values indicated by error bars. The data were from two experiments, each run in duplicate.

On the other hand, for a broad spectrum of sesquiterpene lactones, our research on sesquiterpene lactones of the guaianolide type revealed compounds with immunomodulatory/stimulatory action with no or very low cytotoxicity in macrophages [14,15,16,17,18,19,20,28,29]. These sesquiterpene lactones are known as effective SERCA inhibitors, most notably thapsigargin (Tg) [14]. In the present study, we examined selected guaianolides (**1**–**13**) that are structurally related to thapsigargin (Tg) and trilobolide (Tb). These compounds had not yet been examined for any immunomodulatory effects. Together with other related sesquiterpenoids (**14**–**18**), our screening revealed the ability of some compounds to inhibit NO production in primary murine macrophages. The results are presented in Figure 5. The results of the IC_50_ determination for both guaianolide and germacranolide compounds **1**–**21** are summarized in Table 1.

Seven of the eighteen compounds tested significantly inhibited the in vitro IFN-γ + LPS-activated production of NO (Figure 5). The NO-inhibitory effects of compounds **14**, **15**, **16,** and **18** were associated with remarkable cytotoxicity. It is, however, still too early to say whether such compounds have any potential to be used as immunomodulators. More interestingly, compounds **1**, **13,** and **17** (i.e., 8-epiisoamberboin, 8-deoxylactucin and eupatolide) elicited a significant NO-inhibitory effect (Figure 5) without any suppression of cell viability (Figure 4). These compounds reduced NO production to about 25% compared to controls (Figure 5 and Table 1) in the presence of compound **1.** This result constitutes the first evidence of the immunomodulatory activity of 8-epiisoamberboin (**1**).

A substantial reduction in NO production under inflammatory cellular conditions was also observed for 8-deoxylactucin (**13**). A dose effect study showed that the concentration needed to inhibit NO production by 50% (IC_50_) was 2.81 µM. Compound **13** is a bitter sesquiterpene lactone traditionally extracted from chicory (*Cichorium intybus* L., Asteraceae) roots. The main medicinal interest in this agricultural crop has been the treatment of parasitic diseases [47]. Our recent results showing the inhibitory effect of 8-deoxylactucin (**13**) on NO production in murine peritoneal cells are consistent with those of an earlier study [48]. The authors demonstrated an inhibition of nitric oxide production by this substance, with an IC_50_ of 13 µM in RAW264.7 macrophages. Recently, a non-toxic supercritical-fluid extract containing compounds structurally similar to the lactucin series showed the ability to decrease iNOS expression in a model of inflamed intestinal mucosa [49].

The third compound with an inhibitory effect on NO production was eupatolide (**17**), with an IC_50_ value of 4.38 µM. Our result obtained on peritoneal macrophages is in close agreement with that of several studies performed on the murine macrophage line RAW264.7. In all these cases, eupatolide (**17**) exhibited inhibitory potency against NO production in the concentration range of 1.54–6.43 µM [50,51,52].

Because of the low cytotoxicity of germacranolides **19**–**21**, experiments were conducted to collect information on NO production. All of the results are summarized in Table 1. Parthenolide (**19**) and 9-hydroxyparthenolide (**20**) substantially decreased the production of NO to a level comparable with those of (**13**) and (**17**) in stimulated primary macrophages. Parthenolide (**19**) is a substance with strong therapeutic potential [53]; further research is highly desirable. On the other hand, knowledge about the biological effects of its derivative **20** is very limited. We are probably the first group to reveal new information about the effects of this substance on immune cells.

Laserolide (**21**), which has an angeloyl group attached to its lactone ring, showed partially remarkable activity, which distinguishes it from the common simple methyl lactone or even the exomethylene lactone-containing sesquiterpenes. Substances with hydroxyl or ester groups attached to the lactone ring in this way show the best results in our research so far. These earlier results [16] were obtained by experiments conducted with derivatives of thapsigargin (Tg) [14,15] or trilobolide (Tb) [16,17,18,28] and also archangelolide [19].

### 2.3. Prostaglandin E_2_ Production and Cytokine Secretion

**Figure 6:**
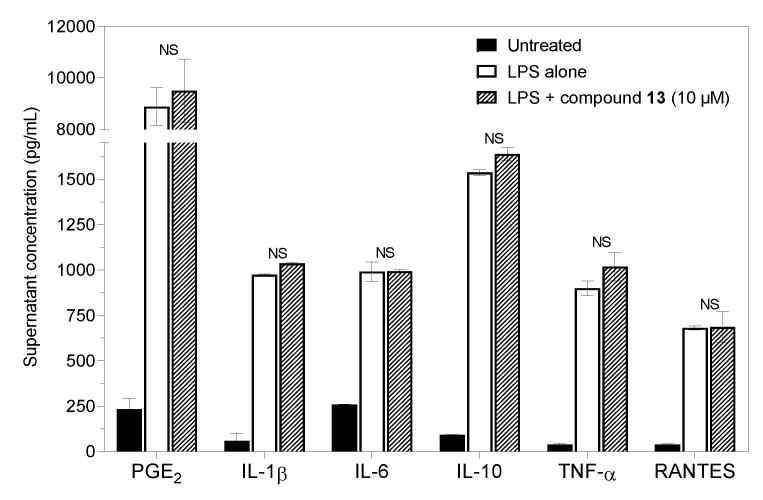
Effects of compound **13** (8-deoxylactucin) on the production of PGE_2_ and cytokines by murine peritoneal cells stimulated with LPS. The cells (2.0 × 10^6^/mL) were cultured for 6 h or 21 h. Columns represent means with S.E.M. values indicated by error bars; NS—statistically non-significant difference.

Based on the clear suppressive effect of 8-deoxylactucin (**13**) on NO production, we expected some anti-inflammatory activity and changes in the secretion of cytokines and prostaglandins. However, compound **13** inhibited only NO and did not interfere with the in vitro immune-activated production of PGE_2_ or with the secretion of cytokines (Figure 6).

## 3. Experimental Section

### 3.1. Chemicals

The substances selected for this investigation came from our own resources. They were processed and later stored during the course of our previous studies, as documented by the references indicated in Table 1.

#### General Methods

Prior to testing, the purity of each selected substance was checked using thin-layer chromatography (TLC) on silica gel plates. When necessary, the compounds were purified by column chromatography (CC) using a short column filled with silica gel. The mobile solvent systems for TLC and CC were chosen based on previously proven procedures developed during the original isolation of the substances from their plant sources, as summarized in references listed in Table 1. To confirm the structural identity of the selected compounds, their MS and ^1^H-NMR spectra were measured again.

### 3.2. Biological Assays

Selected sesquiterpene and exomethylene lactones of the guaianolide and the germacranolide types, isolated and structurally identified in our laboratories, were screened for potential immunomodulatory effects.

#### 3.2.1. Compounds

The tested compounds were dissolved in dimethyl sulfoxide (DMSO) to make stock solutions at a concentration of 50 mM, and then stored at −20 °C. Their biological activities were screened using a final concentration of 10 µM. The concentration of DMSO in biological samples did not exceed 0.02%. This concentration of DMSO was devoid of any effects in biological assays.

#### 3.2.2. Animals and Cell Culture

Female mice of the inbred strain C57BL/6, 8–10 weeks old, were purchased from Charles River Deutschland (Sulzfeld, Germany). They were kept in transparent plastic cages in groups of ten. Lighting in the animal house was turned on from 6 am until 6 pm, keeping the temperature at 22 °C.

The animals were killed by cervical dislocation and injected intraperitoneally with 8 mL of sterile saline. Pooled peritoneal cells collected from the mice (n = 4–6 in individual experiments) were washed and re-suspended in a culture medium. The cell suspension was seeded onto 96-well round-bottom microplates (Costar, Corning, NY, USA). Final samples were supplemented to 100 µL volumes, containing 1–2 × 10^6^ cells per well, depending on the assay. Cultures were maintained at 37 °C, 5% CO_2_ in a humidified incubator (Heraeus Holding GmbH, Hanau, Germany). The complete RPMI-1640 culture medium (Sigma-Aldrich, St. Louis, MO, USA) contained 10% heat-inactivated fetal bovine serum, 2 mM L-glutamine, 50 µg/mL gentamicin, and 5 × 10^−5^ M 2-mercaptoethanol (all Sigma-Aldrich).

All experimental procedures and animal welfare conditions were approved by the respective institutional ethics committees. 

#### 3.2.3. Cytotoxicity Assay

The viability of mouse peritoneal cells was determined using a colorimetric assay based on the cleavage of tetrazolium salt WST-1 by mitochondrial dehydrogenases in viable cells (Roche Diagnostics, Mannheim, Germany). The cells (1 × 10^6^/mL) were cultured as described above. The viability of primary murine macrophages was tested in the presence of 10 µM of the respective test substance for 21 h. After the first 24 h of cultivation, WST-1 was added (10 µL), and the cells were kept in a Heraeus incubator at 37 °C for an additional 3 h. Triton X-100 (1%) was used as a common reference in the cytotoxicity assay. Optical density was evaluated at wavelengths of 450–690 nm.

#### 3.2.4. Nitric Oxide Assay

High-output NO production was induced by a mixture of lipopolysaccharide (LPS from *E. coli* 0111:B4, 0.1 ng/mL; Sigma) and murine recombinant interferon-γ (IFN-γ, 5 ng/mL; R&D Systems, Minneapolis, MN, USA) in mouse peritoneal cells (2 × 10^6^/mL). The tested compounds were applied concomitantly with both priming stimuli, and peritoneal macrophages were cultured for 24 h. The concentration of nitrites in the supernatants of the cell cultures was taken as a measure of NO production. It was detected in individual cell-free samples (50 µL) incubated for 5 min at ambient temperature with an aliquot of Griess reagent (1% sulfanilamide/0.1% naphtylendiamine/2.5% H_3_PO_4_). Absorbance at 540 nm was recorded using a microplate spectrophotometer (Tecan, Grödig, Austria). A nitrite calibration curve was used to convert absorbance values to µM of nitrite.

#### 3.2.5. Prostaglandin E_2_ and Cytokine Assays

The concentration of PGE_2_ was determined in the supernatants of cells cultured (2 × 10^6^/mL) after the addition of 10 ng/mL LPS for an interval of 6 h. The levels of cytokines TNF-α, IL-1β, IL-6, and IL-10, and the chemokine RANTES were determined in the supernatants of cells cultured (2 × 10^6^/mL) in the presence of 1 µg/mL LPS for an interval 21 h. The extent of PGE_2_ release and cytokine secretion was determined using enzyme-linked immune-absorbent assay (ELISA) kits according to the manufacturer’s instructions (R&D Systems, Minneapolis, MN, USA).

#### 3.2.6. Statistical Analysis

The data were analyzed using analysis of variance (ANOVA) with subsequent Dunnett’s multiple comparison test, and the data were graphically visualized using the program Prism 6.05 (GraphPad Software, San Diego, CA, USA).

## 4. Discussion

The other results of this study were somewhat surprising. In one set of assays, the experiments were repeated with samples prepared from the same animals as for the NO analysis. Based on our experience with studying anti-inflammatory effects in murine macrophages, the concentrations of LPS necessary to stimulate an increase in PGE_2_ and cytokine secretion measured with substance **13** were high enough to demonstrate a possible inhibitory effect. It is generally accepted that the α,β-unsaturated carbonyl moieties, such as those found in exomethylene lactones, are essential for causing anti-inflammatory, analgesic, cytotoxic and other effects. There are several possible explanations for these results. For example, the time of the detection of PGE_2_ and cytokine secretion could have been shifted in a way that was not anticipated. In addition, some differences in the results may have been due to the origin of the substances. A few publications have studied 8-deoxylactucin, which was not commercially available until recently. Instead of the pure compound, chicory extract was applied to the cellular models in most of these studies. The data other researchers obtained were from immune or non-immune cell lines or during in vivo testing [48] and not from primary immune cells. However, the details of the experimental conditions, including the concentrations of the extracts/substances used and the time of incubation, varied [48,54,55], so the results of the anti-inflammatory assays are not easy to compare. In many articles, anti-inflammatory/immunomodulatory activity was examined through molecular assays at the gene expression or transcription factor level, but the end products (production and concentration of cytokines and prostaglandins) were not determined. Our finding of the inhibition of NO production without a direct correlation with decreased levels of cytokines and PGE_2_ in the presence of 8-deoxylactucin (**13**) is supported by a review [56], which showed that the measurement of NO alone is sufficient for the initial screening for possible anti-inflammatory action in a model of LPS-induced macrophages. In the present work, we did find 8-deoxylactucin (**13**) to have the potential to inhibit NO production. The extent of this NO-inhibiting and anti-inflammatory potential, however, remains to be clarified in further research. The recent commercial availability of 8-deoxylactucin may help shed some light on the topic.

## 5. Conclusions

Two series of sesquiterpenoids (selected guaianolides and germacranolides), which had not yet been examined for any immunomodulatory effects, were screened in this study. Our screening revealed the abilities of some of the tested compounds to inhibit NO production in LPS-stimulated primary murine macrophages. In the group of tested guaianolides, a significant decrease in NO production was observed with 8-epiisoamberboin (**1**), which contains only a methyl-lactone moiety. Another compound of particular interest was 8-deoxylactucin (**13**), which had a significant inhibitory effect on NO production without any impact on cell viability, even though it contains an activated α-exomethylene group in its lactone moiety. These two substances with potential pharmacological uses therefore merit further investigation.

## Figures and Tables

**Table 1:** Inhibition of nitric oxide production (IC_50_) in the presence of compounds **1**–**21** in (LPS + IFN-γ)-activated mouse peritoneal cells. IC_50_ values were obtained by testing concentrations of 0.01–50.0 μM (micromole) for each substance.

Compound	IC_50_ (µM)	95% Confidence Interval	References *
**1**. 8-epiisoamberboin	23.76	16.72–33.76	[30]
**2**. 8-epiisolippidiol	>100	–	[31,32]
**3**. deacetylmatricarin	>100	–	[33]
**4**. 11β,13-dihydrolactucin	>100	–	[34]
**5**. 4,8-diepiisolippidiol glucoside	>100	–	[34]
**6**. deacetylmatricarin glucoside	>100	–	[33]
**7**. crepidiaside B	>100	–	[35,36]
**8**. ixerin F	>100	–	[37]
**9**. 11β-hydroxyleukodin glucoside	>100	–	[38]
**10**. 11β-hydroxydeacetylmatricarin glucoside	>100	–	[33]
**11**. 11,13-dehydro-8-epiisolippidiol glucoside	>100	–	[39]
**12**. ixerin D	>100	–	[40]
**13**. 8-deoxylactucin	2.81	2.08–3.79	[34,35]
**14**. cynaropicrin	9.87	3.99–24.39	[20,41]
**15**. helenalin	0.74	0.45–1.22	[29,42]
**16**. costunolide	3.20	2.51–4.08	[43]
**17**. eupatolide	4.38	2.85–6.73	[43,44]
**18**. eupatoriopicrin	1.65	0.76–3.59	[43,44]
**19**. parthenolide	2.79	1.84–4.22	[45]
**20**. 9-hydroxyparthenolide	5.64	3.09–10.27	[45]
**21**. laserolide	90.16	1.07–2.61	[16,46]

* References listed in this column refer to plant sources and to the isolation and chemical identity of compounds **1**–**21**.

## Data Availability

Data presented in this study are available in the article.
